# Statistics of the Medical Profession in the United States

**Published:** 1854-09

**Authors:** C. A. Lee


ART. III.—*Statistics of the Medical Profession in the United States.* By C. A. Lee, M. D.

By the census of the Unlted States of 1850, we learn that there are 40,564 physicians in the United States. Whether this includes the irregular practitioners and quacks we are not informed, but presume that it does not. The reader will perceive, by consulting the following table, the number of practitioners in the U. States and in each state of the Union, the ratio of practitioners to the population, and the number of square miles in each state and territory:

| STATES. | No. of Physicians. | Population. | Ratio of Physicians to population. | No. of Inhabitants to square mile. |
|---|---|---|---|---|
| Maine, | 659 | 583,169 | 854 | 12.52 |
| New Hampshire, | 623 | 317,976 | 510 | 39.6 |
| Vermont, | 663 | 314,120 | 473 | 39.26 |
| Massachusetts, | 1643 | 994,514 | 605 | 137.17 |
| Rhode Island, | 217 | 147,545 | 679 | 122.95 |
| Connecticut, | 560 | 370,792 | 662 | 78.06 |
| New York, | 5060 | 3,097,394 | 612 | 67.33 |
| New Jersey, | 608 | 489,555 | 801 | 71.46 |
| Pennsylvania, | 4071 | 2,311,786 | 570 | 49.19 |
| Delaware, | 114 | 91,532 | 838 | 43.17 |
| Maryland, | 990 | 583,034 | 588 | 53.00 |
| District Columbia, | 104 | 51,687 | 496 | 1033.74 |
| Virginia, | 2163 | 1,421,661 | 657 | 23.17 |
| North Carolina, | 1083 | 869,039 | 802 | 19.1 |
| South Carolina, | 905 | 668,507 | 738 | 23.87 |
| Georgia, | 1295 | 906,185 | 699 | 15.62 |
| Florida, | 135 | 87,445 | 647 | 1.48 |
| Alabama, | 1264 | 771,623 | 610 | 15.21 |
| Mississippi, | 1217 | 606,526 | 498 | 12.86 |
| Louisiana, | 912 | 517,762 | 567 | 12.52 |
| Texas, | 616 | 212,592 | 346 | 0.65 |
| Arkansas, | 449 | 209,897 | 465 | 4.02 |
| Tennessee, | 1523 | 1,002,717 | 658 | 22.79 |
| Kentucky, | 1818 | 982,407 | 540 | 26.07 |
| Ohio, | 4263 | 1,980,329 | 464 | 49.55 |
| Michigan, | 854 | 397,654 | 465 | 7.07 |
| Indiana, | 2170 | 988,416 | 455 | 29.24 |
| Illinois, | 1402 | 851,470 | 607 | 15.37 |
| Missouri, | 1351 | 682,044 | 504 | 10.49 |
| Iowa, | 542 | 192,214 | 354 | 3.77 |
| Wisconsin, | 581 | 305,391 | 525 | 5.66 |
| California, | 626 | 92,597 | 147 | 0.49 |
| TERRITORIES. | | | | |
| Minesota, | 13 | 6,077 | 467 | 0.04 |
| New Mexico, | 9 | 61,547 | 6838 | 0.29 |
| Oregon, | 45 | 13,294 | 295 | 0.04 |
| Utah, | 16 | 11,380 | 711 | 0.06 |
| Total, | 40,564 | | | |

The total population is 23,191,876; the ratio of practitioners to the whole population is one to 571. California is better supplied with physicians than any other part of the United States, there being one to every 147 inhabitants, while New Mexico is the most deficient, there being but one to 6,838 inhabitants. Considering the sparseness and thinly scattered state of the population, in most parts of the Union, it may well admit of doubt, whether the surplus is very great. That it is so in the older and comparatively more settled states is sufficiently obvious.

The mortality among medical practitioners is greater than among any other class of men; we believe, taking the whole United States, that it will not fall short of one in twenty-five, annually. This will give a total of 1622 deaths, which are to be supplied from our medical graduates. Considering that large numbers of young men, especially from the Southern States, are educated to the profession, without any view to practice, and that many more enter upon other pursuits, and engage in other and more profitable callings, the number of those who annually graduate from our medical colleges will not appear disproportionately large; we do not know the exact number, but suppose it will not vary far from 2000.

